# Impact of Innovative Technologies on the Content of Vitamin C and Its Bioavailability from Processed Fruit and Vegetable Products

**DOI:** 10.3390/antiox10010054

**Published:** 2021-01-05

**Authors:** Monika Mieszczakowska-Frąc, Karolina Celejewska, Witold Płocharski

**Affiliations:** Department of Fruit and Vegetable Storage and Processing, Research Institute of Horticulture, 96-100 Skierniewice, Poland; Karolina.Celejewska@inhort.pl (K.C.); Witold.Plocharski@inhort.pl (W.P.)

**Keywords:** vitamin C, fruit, vegetable, food processing, non-thermal technology, degradation, bioavailability

## Abstract

Nowadays, thermal treatments are used for extending the shelf-life of vegetable and fruit products by inactivating microorganisms and enzymes. On the other hand, heat treatments often induce undesirable changes in the quality of the final product, e.g., losses of nutrients, color alterations, changes in flavor, and smell. Therefore, the food industry is opening up to new technologies that are less aggressive than thermal treatment to avoid the negative effects of thermal pasteurization. Non-thermal processing technologies have been developed during the last decades as an alternative to thermal food preservation. Processing changes the structure of fruit and vegetables, and hence the bioavailability of the nutrients contained in them. In this review, special attention has been devoted to the effects of modern technologies of fruit and vegetable processing, such as minimal processing (MPFV), high-pressure processing (HPP), high-pressure homogenization (HPH), ultrasounds (US), pulsed electric fields (PEF), on the stability and bioavailability of vitamin C.

## 1. Introduction

These days people know that fruits and vegetables are rich in antioxidant compounds such as vitamin E, C, and carotenoids with provitamin A properties, and it is considered that they reduce the risk of chronic diseases by protecting against free-radical mediated damage [1]. However, it refers to the last 100 years when our knowledge was broadened enough to know the causes and remedies of many diseases that haunted humanity, including those associated with a not adequate supply of vitamin C.

The first research on vitamin C (L-Ascorbic acid (L-3-keto-thero-hexuronic acid-γ-lactone, Formula 6.18, I)) dates back to 1928 when people were looking for treatment options for scurvy [2,3,4]; therefore, vitamin C is called ascorbic acid differently from the French name (Fr. ascorbine, abbrev. A (nti) -scorb (ut) -ine)). For centuries people, especially sailors and polar investigators, were subjected to the disease scurvy, which is characterized by apathy, weakness, easy bruising with tiny or large skin hemorrhages, friable bleeding gums, and swollen legs [5]. The history of scurvy could finally close. Now we know that scurvy can be prevented with an intake of 10 mg vitamin C/day [6]. However, vitamin C plays a role not only in scurvy development, but it is necessary for the proper functioning of the human body as an enzyme cofactor for biochemical reactions catalyzed by monooxygenases, dioxygenases, and mixed-function oxygenases. In the European Union, a list of permitted health claims was elaborated [7]—there are 15 claims concerning vitamin C, more than for any other vitamin. Among others, it is stated that ascorbic acid contributes to maintaining the normal function of the immune system, contributes to proper collagen formation, necessary for normal functioning of blood vessels, the function of bones, cartilage, gums, skin, and teeth, and contributes to the protection of cells from oxidative stress.

Vitamin C is an exogenous compound for humans, meaning that it is not produced in the human body and must be supplied in food. The daily referenced intake of vitamin C is on the level 80 mg/day [8], whereas German, Austrian, and Swiss nutrition societies consider that a reference value (recommended intake) should be 110 mg/day for men and 95 mg/day for adult women. However, these recommendations may differ depending on gender, age, and health condition [9]. The greatest amounts of vitamin C can be found in fruits and vegetables. This vitamin is particularly abundant in rose hips, black currants, sea buckthorn, strawberries, kiwifruit, parsley, oranges, lemons, grapefruit, papaya, pineapple, mango, quince, a variety of cabbages, broccoli, cauliflower, peppers, turnip, kale, and potatoes [10,11]. In Table 1 and Table 2, there are listed fruit and vegetables considered to be the best source of vitamin C.

Vitamins are minor, but essential food ingredients. Deficiency can result in hypovitaminosis and, if more severe, avitaminosis. Hence, it is very important to preserve them during the storage and processing of fruit and vegetables [11].

A large part of the species of fruit and vegetables are available only for a short time, just after harvesting. Developed storage technologies make it possible to keep both fruit and vegetables fresh for many months. Nevertheless, since the old days, most fruit and vegetables are processed, which allows the full use of the harvest and the consumption of fruit and vegetable products throughout the year. Processing is an excellent possibility to extend the shelf life, but on the other hand, it is associated with significant losses of many pro-health ingredients, including vitamin C, which is the least stable of all vitamins and is easily destroyed during processing. Very often, processing involves the use of high temperatures, aeration, exposure to light and oxygen, and these are the main factors causing adverse changes in the composition of processed fruits and vegetables [13,14,15,16,17,18,19,20,21]. These factors promote the oxidation of L-ascorbic acid to unstable L-dehydroascorbic acid [22], which, as a result of hydrolysis and opening of the lactone ring, transforms into vitamin-inactive 2,3-diketogulonic acid [23].

Vitamin C is characterized by low thermal stability and a tendency to oxidize easily; therefore, each process using elevated temperature causes loss of this vitamin compared to fresh material. These losses can be from 20% to even 90% depending on the temperature level, the duration of the processing operation, and contact with oxygen.

It is generally observed that when vitamin C is well retained, other nutrients are usually also well retained [18,24,25]. Therefore it is important to follow how particular processing operations influence the quantity of this vitamin and to which extent it is detrimental for the final product quality.

The impulse for the creation of the review was the extension of the current nutritional trend and the expectations of consumers looking for products with high vitamin and/or antioxidant value, as well as intensively developing processing technologies. The purpose of this review is to summarize the impact of innovative low-temperature techniques on antioxidants (as exemplified by vitamin C) in the processing of fruit and vegetables compared to traditional, high-temperature processing.

## 2. Traditional Processing—Critical Points

The precise amount of vitamin C in a specific food varies according to the season of harvesting, transport, storage time before use, and processing practices. Even minor operations related to the processing of fruit and vegetables, such as peeling, cutting, or crushing, can change the matrix structure and activate ascorbic acid oxidase, which contributes to the oxidation of L-ascorbic acid, resulting in the huge loss of this valuable vitamin [26].

### 2.1. Low Temperature—Freezing Technology

The best method of preservation of vitamin C in fruit and vegetables is freezing. Quick post-harvest freezing of raw materials contributes to the preservation of this ingredient in the products. Some vegetables are blanched before freezing in water or steam, which may decrease the content of water-soluble compounds; however, it also inactivates enzymes in the raw material. Li et al. [27] indicated that in some cases, fresh products, stored for up to several days, had lower nutrient content than analogous frozen products. When the fruit is not blanched before freezing, then oxidation of ascorbic acid occurs at a slow rate during frozen storage.

A comprehensive review of the changes of ascorbic acid in fruits and vegetables during processing by conventional methods such as freezing and canning has been given by Rickman and colleagues [28]. The authors pointed out that the effects of processing, storage, and cooking are highly variable by commodity. The initial thermal treatment of processed products can cause loss of water-soluble and oxygen-labile nutrients such as vitamin C and B vitamins. However, these nutrients are relatively stable during subsequent canned storage owing to the lack of oxygen. Frozen products, in turn, lose fewer nutrients initially because of the short heating time in blanching, but they lose more nutrients during storage owing to oxidation. The general conclusion of this review was that while canned foods are often perceived as less nutritious than fresh or frozen products, the research reveals that this is not always true.

Another study [29] showed, by the example of fruit and vegetables (lemon, cranberry, apple, red pepper, broccoli, and sweet potatoes) that not only high temperature adversely affects the stability of vitamin C, but also low freezing temperatures. Samples were frozen for one week at a temperature of −16 °C and lost about 30% of the content of vitamin C, while when cooked for 15 min, more than 50%.

### 2.2. High Temperature—Pasteurization, Sterilization, Blanching, Cooking, Steaming, etc.

The use of high temperatures in the processing of fruit and vegetables plays a very important role, allowing the product to be microbiologically safe, but also causes changes in ingredients—e.g., protein denaturation, starch gelatinization, which in turn increases the digestibility of processed fruit and vegetables. On the other hand, the use of high temperatures negatively affects many health-promoting ingredients, especially vitamins. However, both pasteurization (temperature, usually less than 100 °C) and sterilization (temperature, 110–121 °C) are still considered as the most reliable methods of product preservation, guaranteeing long shelf life and product safety.

The impact of thermal processing (heating at 92 °C for 10 min, then grinding, the mixture was passed through a sieve and concentrated under a pressure of −0.96 bar at 65 °C, pasteurized at 100 °C for 10 min) and the lyophilization of red and yellow tomatoes was studied [30]. The vitamin C losses were high (about 80%) in the processing of both red and yellow tomatoes.

Klopotek et al. [31] showed that the greatest losses of vitamin C in the production of strawberry juice were caused by pressing and pasteurization. The pressing process resulted in a loss of vitamin C of about 22%, while pasteurization at high temperature (85 °C) reduced the content of ascorbic acid by 35% compared to the filtered juice. The lower losses of vitamin C were observed by authors during strawberries processing to puree, about 12% as compared to raw strawberries. Pasteurization of blackcurrant nectar (80 °C for 27 s in a plate heat exchanger) resulted in a loss of 2–6% of the vitamin C content [32]. In contrast, sterilization of fruit and vegetable products results in a loss of vitamin C at the level of 51–56% [11].

El-Ishaq and Obirinakem [4] demonstrated in their work that pasteurization at a lower temperature (40 °C) on fruit juices (pineapple, orange, watermelon, and tomato juices) also resulted in a loss of vitamin C at the level of 39–47%, regardless of the initial content of L-ascorbic acid.

High temperature is not the only contributing factor that causes significant losses of ascorbic acid in the production of fruit juices. Often, the technology includes an enzymatic process for high-pectin-rich fruits to increase juice yield. The enzymatic treatment of fruit mash can also cause significant losses of ascorbic acid, which was also observed by Mieszczakowska-Frąc et al. [33]. During the production of blackcurrant juices, the fruit mash was treated with pectinolytic preparations for 1 h (enzyme dose 200 g/t), the loss of vitamin C at this technological stage was at the level of 26–31%, depending on the enzyme preparation used.

Among the vegetables that significantly enrich the diet with vitamin C are broccoli, which is usually cooked for a short time. However, even short treatments can contribute to a significant loss of vitamin C: 0.5 min—loss of about 19%, 1.5 min—47%, and at 5 min, as much as 66% [34]. Also, steaming, boiling, and sous-vide processing of broccoli significantly reduced the vitamin C content [35]. The research conducted on carrot blanching showed that the lowest losses of vitamin C took place when the vegetables were heat-treated at high-temperature for a short time (e.g., 98 °C for 1 min) than at low temperature for a longer time (60 °C for 40 min) [13]. Losses of L-ascorbic acid at these blanching treatments were 15% and 99%, respectively. Generally, blanching can inactivate ascorbic acid oxidase, thus slowing down the degradation rate of vitamin C [36].

The experiment carried out on the effect of different cooking methods (boiling, blanching, steaming, and microwaving) on the content of vitamins in vegetables (broccoli, chard, potato, sweet potato, carrot, spinach, and zucchini) showed that boiling significantly reduced vitamin C content in all samples in the range of 26–100%. The retention of vitamin C after blanching treatment ranged from 58% to 89%, after steaming ranged from 0% to 89%. Microwaving had less of an impact, and retention was in the range of 67–112% [37].

The use of the same thermal treatments but on different species of vegetables resulted in very differentiated degradation of vitamin C. Popova [38] used food processing like cooking, steaming, and microwaves on cauliflower, pepper, potatoes, carrots, cabbage, and eggplant. In this study, boiling destroyed most of vitamin C (27% to 69%) in all the samples, while the greatest losses were observed in red pepper and the lowest in potatoes. Among the examined processes, the highest losses of ascorbic acid were observed under the influence of microwaves, and the smallest degradation of vitamin C occurred when vegetables were steamed.

In order to minimize the loss of thermolabile ingredients, new methods were introduced into production, such as aseptic packaging. In this technology, the products are paced into aseptic packages in aseptic conditions, which enables the use high temperatures for a very short time (HTST method—high-temperature short time). Such an attitude makes the loss of nutritional value is lower when compared with hot filling. However, high temperature applied even for a very short time (HTST) often causes unfavorable changes in color and aroma [39].

### 2.3. Drying Technology

Drying is one of the oldest methods used to extend the shelf life of fruits and vegetables and comprises an important part of food processing. It consists of removing part of the water from the material by evaporating it, with the heat supplied from the outside [40]. The most common drying agent is hot air. Current convective drying is used on a large scale in the food drying industry due to economic reasons and a well-known and controlled process. The disadvantage of this method is that it may cause an adverse effect on fruit quality [17,25,41,42,43,44,45]. Convective drying is usually a long-lasting process, and high temperature used can lead to a significant reduction of a dried product’s nutritional and sensorial quality induced by chemical, physical and biological reactions [19,46,47,48]. A good alternative for convective drying can be freeze-drying, which is considered to be the best method in terms of preserving final product quality [25]. In general freeze-dried samples: blueberries, sour cherries, cranberries, and strawberries [44] were characterized by the highest retention of vitamin C when compared to other methods, irrespectively of the fruit species, whilst air-drying resulted in the lowest level of this compound, which confirmed generally known trend.

Ali and colleagues [25] studied the influence of the drying method (microwave drying, oven drying, sunlight drying, and freeze-drying) heat supply, light and oxygen exposure, and drying time on the vitamin C level of guava slices. They found that the key parameters affecting the ascorbic acid concentration in guava are drying temperature and drying time. Freeze-dried samples had the highest ascorbic acid content and the best color quality. In contrast, the lowest retention of ascorbic acid, below 10% of fresh fruit content, was found in sun-dried guava slices. It confirms that ascorbic acid is very sensitive to sunlight, oxygen, and drying time. Moreover, in comparison, the parameters of oven treatments (50 °C for 18 h; 60 °C for 15 h; 70 °C for 11 h; 80 °C for 4.5 h; 90 °C for 3.5 h) indicated that short drying duration preserves more ascorbic acid than the usage of low drying temperature use.

Another author’s team [42] working on papaya fruit, investigated the degradation of vitamin C during hot air drying at a temperature range from 40 to 70 °C and two levels of air velocity (1 and 1.32 m/s). Lower air temperature (40 °C and 50 °C) and lower velocity induced higher retention of this nutrient (about 52% and 49%, respectively) at the end of drying, while at a temperature of 60 and 70 °C ascorbic acid retentions were only 34% and 24%, respectively. This pattern was also observed for runs carried out at 1.32 m/s, when nutrients retentions were: 49%, 52%, 42%, and 38% at an air temperature of 40, 50, 60, and 70 °C, respectively. Thermal damage of a product during drying was directly proportional to the processing time: the longer the remaining time in the dryer, the longer exposure time for fruits with hot air was obtained, and consequently, the higher nutrient degradation occurred. These relationships are also confirmed by studies conducted on another plant material: tomatoes [16], wild rose [17], cranberries [41], strawberry [20], and sour cherries [49]. In a study carried out with strawberries, a particularly visible effect of drying time on the retention of vitamin C was noted at the highest tested temperature of 70 °C, where after 1 h of the process, the retention was about 90% and after 7 h of drying only 40% of ascorbic acid was left.

## 3. Consumers Expectations of Food

More and more consumers are conscious of the importance of nutrition in maintaining good health. They expect food with an extended shelf life, but also above all with improved quality and naturalness. In addition, they expect natural supplementation with pro-health compounds and their high bioavailability from food consumed.

Unfortunately, virtually every attempt to process fruit involves the loss of bioactive ingredients. The processing industry is focused on looking for new solutions that allow not only to increase production efficiency but also to improve the quality of fruit and vegetable products so that, while maintaining their natural values, they are able to meet the expectations of an increasingly aware and demanding consumer. Initially, efforts were made to develop optimal and minimal exposure times for processed products to temperature (high temperature, short time). For example, to achieve the most valuable attributes, the juice was minimally processed using cold-pressing to reduce temperature and oxygen exposure, but this type of extraction allowed obtaining a product with only a few days of shelf-life [50,51].

Currently, the state of knowledge indicates that replacing the classic thermal operations used at various stages of the production of fruit and vegetable preserves (e.g., raw material pre-treatment, pasteurization, blanching, and sterilization) with innovative non-thermal methods may contribute not only to increase the efficiency of the processing (e.g., the yield of juices pressing) but also reduce the loss of labile bioactive ingredients and increase their stability during product storage [52,53].

## 4. Innovative, Non-Thermal Technologies in Fruit and Vegetable Processing

Non-thermal technologies are seen as very effective alternatives for preserving products with the ability to keep their nutritional and sensory value as high as possible [54].

### 4.1. High-Pressure Processing (HPP)

High-pressure processing (HPP) (also known as high hydrostatic pressure (HHP)) or ultra-high pressure (UHP)—a method that usually uses a pressure of 600 MPa. The HPP method in the food industry was first used in Japan to extend the shelf life of citrus juices and during the production of fruit jams [55]. Currently, this method has also been introduced in the production of juices in the USA and European countries.

High-pressure treatment not only increases microbiological stability and reduces the enzyme activity in processed products, but also, compared to traditional thermal processing, results in better retention of bioactive compounds [56,57]. The application of high pressures is carried out at low temperatures; therefore, this process enables better preservation of bioactive ingredients, including vitamin C.

According to Koutchma and colleagues [39], HPP is one of the simplest and environmentally friendly ways to destroy microbiological pathogenies. HPP technology is currently commercial serves as the primary processing method in the cold-pressed juice market. The product is subjected to high pressure when it is already packed in the final packaging. The applied pressure can range from 200 to 900 MPa. The physical pressure compresses the product uniformly, which leads to a reduction in the number of microorganisms while keeping quality parameters unchanged; however, the minimum pressure required to inactivate vegetative microorganisms at ambient temperatures is 400 MPa [58,59]. The temperature used for HPP is usually below 40 °C, and the time used for the process ranges from a few minutes to an hour. 

In the comparison presented by Koutchma et al. [39], the preservation of vitamin C when using different HPP conditions of 18 fruit juices from various fruit species ranged from 76% to 96%. The largest vitamin C losses at 24% were observed in tomato juice using 500 MPa at 25 °C for 10 min [60] and carrot juice using 500 MPa at 40 °C for 15 min [61]. Some studies even indicate an increase in the content of vitamin C (about 3%) after HPP treatment of juice.

Patras et al. [56] compared the content of bioactive compounds, including vitamin C, in strawberry purées, which were subjected to high pressure in the range of 400–500–600 MPa and thermally processed at 70 °C. The research showed significant losses of ascorbic acid at the level of 22.6% in thermally treated purée. This loss was significantly higher than in the high-pressure purées, which showed less than 9% reduction of vitamin C compared to the initial level of vitamin C in the strawberry purées.

The data compiled by Tewari and colleagues [59] shows that most of the conducted studies taking into account the impact of HPP on fruit and vegetable products resulted in a very high vitamin C retention (79–99% in fruit products and 67–93% in vegetable products). Additionally, it revealed better stability of vitamin C during the storage of pressure treated products compared to products after thermal treatment [57,62,63,64,65,66,67,68].

In summary, HPP technology shows a significant conservation potential for vitamin C not only during processing but also during storage. HPP is an interesting alternative to the thermal treatment of food products, allowing both microbiological stability and better preservation of health-promoting compounds.

### 4.2. High-Pressure Homogenization (HPH)

High-pressure homogenization (HPH) or ultra-high pressure homogenization (UHPH) is a promising non-thermal preservation technique that uses pressures in the range of 100–600 MPa, which is much higher than is in traditional homogenization used in the food industry (20–50 MPa). This method is used on liquid products, where the rheological properties of the product change under the influence of high pressures [69,70]. The process allows the solid particles in the suspension to be broken down, thanks to which the product acquires a uniform, smoother consistency [71,72].

Besides, in the process of (ultra) high-pressure homogenization, inactivation of microorganisms [73,74,75,76], and a reduction in enzyme activity takes place [77], which increases the shelf life of the food product, which makes it possible to replace high-temperature methods of preservation (pasteurization) with low-temperature processes. Due to the low processing temperature, the maintenance of the thermolabile compounds is increased. There are also studies confirming the better preservation of bioactive ingredients [72]: vitamins [78] and polyphenols [78,79], including anthocyanins [80], after high-pressure homogenization.

Studies focusing on the stability of bioactive ingredients after the use of HPH indicate the beneficial effects of this non-thermal technological method on the retention of these ingredients. The remaining content of L-ascorbic acid in the ultra-high pressure homogenization treated samples of orange juice at any pressure was significantly higher than in the thermal pasteurized one [78]. The content of L-ascorbic acid in samples treated with lower pressure (100 and 200 MPa) decreased only about 2% and 5%, while the usage of higher pressure (300 MPa) resulted in the reduction of 11%. This was due to an increase in temperature during the HPH process along with increasing pressure to 45 °C, 70 °C, and 94 °C, respectively, and it is known that higher temperature accelerates the oxidation of ascorbic acid.

Very promising results have been obtained with the application of HPH in the production of apple juice [81] when the combinations of pressure (100, 200, and 300 MPa) and inlet temperatures (4 and 20 °C) were assayed. Additionally, apple juice was pasteurized at 90 °C for 4 min as a control. Vitamin C concentrations did not change in ultra HPH treated samples; the values were the same as in raw juice, whilst pasteurized at 90 °C resulted in obtaining a juice containing only 11% of the initial level of vitamin C. Other studies also confirm the absence of negative changes in vitamin C content in fruit and vegetable products subjected to HPH, e.g., orange juice [77], tomato paste [82], pomegranate juice [83]. 

There are also studies [84] showing that high-pressure homogenization of rosehip juice in a pressure range of 75–125 MPa resulted in a loss of ascorbic acid of about 20%. In addition, the use of three cycles of the process instead of one cycle increases the loss of ascorbic acid by up to 37%.

### 4.3. Ultrasound (US)

Ultrasound is a form of energy generated by sound waves out of the human audible range, i.e., above 16 kHz. Ultrasounds can be considered as the air vibrations of a frequency from 20 kHz to 100 kHz. Ultrasound, like other acoustic waves, is a mechanical wave that, while traveling through the medium, causes periodical dilution and thickening of the medium and induces local pressure increases and drops. They have the ability to interact with matter and can modify the course of physical or chemical processes [85,86,87,88].

US, acting mechanically with the structure of the medium, causes physical destruction of the cells of the walls of the soft and hard parts of the fruit; thus, contributing to increase the efficiency of extraction of juice and bioactive ingredients from the matrix and also promote stability of these components during storage. This effect entails a shortening of the duration of the necessary thermal treatments, a reduction in the amount of oxygen dissolved in the material, and the ability of ultrasound to inactivate native enzymes. In addition, ultrasonic waves have an antibacterial effect [89,90,91,92], and hence their use is being considered as an alternative to the high-temperature preservation of juices [93], that is, pasteurization, which causes significant losses of bioactive components.

Ultrasound techniques are considered to be environmentally friendly and are increasingly being used in various areas of the economy, including the food industry [89,94,95].

Aadil et al. [96] investigated the effect of ultrasound on the quality of grapefruit juice. The pressed juice was sonicated for 30, 60, and 90 min. (US parameters: 28 kHz frequency and temperature of 20 °C). The results showed a significant increase in vitamin C in all the sonicated juice samples as compared to the control (no US-treatment juice). There was an increase of 14% after 30 min US to 28% after 90 min of US exposure. A high level of retention of ascorbic acid was observed during strawberry juice sonication with a different range of acoustic energy density. With an increase of time and sonication intensity, retention of ascorbic acid decreased. The largest reduction in ascorbic acid after sonication was less than 15% [93].

Using the example of freshly squeezed mango juice, the effect of high-temperature treatment (at 90 °C) was investigated in comparison to the ultrasonic process lasted a various period of times (15, 30, and 60 min) at 25 °C (40 kHz, 30 W) on the juice quality, including the stability of vitamin C [97]. The authors observed the highest degradation (65%) of ascorbic acid for thermally treated juice when compared to the untreated mango juice. Ultrasound treatment had a much better effect on the retention of L-ascorbic acid than thermal pasteurization. The reduction after sonication for 15 min and 30 min was about 13% and 16%, respectively. Extension of time of US impact to 60 min resulted in an increase in vitamin C loss to 28%. Moreover, a positive effect of the sound waves on the carotene content in mango juice was found. These results agreed with other similar studies on sonicated purple cactus pear juice [90], where the effect of different ultrasound amplitude levels and treatment time (40% and 60% for 10, 15, 25 min; 80% for 3, 5, 8, 10, 15, and 25 min) on the characteristics of cactus juice were evaluated. The content of ascorbic acid in all the treatments was similar to the control sample except for the juice treated at 80% amplitude level for 8 and 15 min, where results were lower by about 25%.

Several studies [92,93,98,99,100,101,102,103] confirmed that this technology allows the achievement of microbiologically juice safety and shelf-life stable juice without compromising the retention of antioxidant compounds.

There is a great deal of literature supporting US application as a feasible method for accelerating fruit dehydration processes [104]. It was established that the application of US during the osmotic dehydration of sour cherry [105] and blueberry fruit [106] resulting in significantly intensified mass transfer.

The ability of US to mechanically interact with the cellular structure of plant tissue seems to offer new possibilities for the drying of plant material. Especially interesting was the expected structure modification in the surface layer, which can lead to possible micro-perforation of the skin, and thus reduce diffusion barriers. Thanks to this effect, US may also be used as an agent for accelerating the final process of drying [107,108,109,110,111,112,113,114].

Despite the enormous popularity of the use of sound waves in the drying process, there is still little information in the literature on the stability of L-ascorbic acid during ultrasound-assisted drying; perhaps due to the accelerated degradation of this antioxidant under the influence of US. Tao et al. [36] studied the effect of water blanching pre-treatment (30 s) and surface contacting ultrasound-assisted (429.3 and 1131.1 W/m^2^) assisted air drying of white cabbage. It was shown that among blanched samples, cabbages dried only by air alone contained significantly higher vitamin C content than cabbages dried with the assistance of sonication. De Silva Junior et al. [115] observed a significant reduction of ascorbic acid content in papaya during drying using four different methods. They reported the lowest losses of this compound (41.3 %) in samples dried with the aid of ultrasound in combination with comparison to vacuum drying, ultrasound-assisted drying, and controls (without ultrasound and vacuum).

### 4.4. Pulsed Electric Fields (PEFs)

Pulsed electric field processing is a non-thermal method in contrast to thermal pasteurization. PEF treatments involve the application of pulses of high voltage (typically above 20 kV/cm) to liquid foods placed between two electrodes [116].

The use of this method for the destruction of microorganisms is based on the generation of oppositely charged cargo around the cell membrane of microorganisms and creating a large potential difference. A potential difference of about 1 V causes the formation of micropores, which lead to the destruction of microorganisms [55,117]. Nevertheless, the use of PEF also generates high temperatures. The PEF method also affects the structure of fruit and vegetables, which is why it is recommended for the intensification of many processes, such as osmotic dehydration, drying, and juice extraction [116,118].

The influence of the pulsed electric field on the content of bioactive compounds, including vitamin C, was tested on the example of watermelon juice [119]. The following process parameters were investigated: electric field strength (30–35 kV/cm), pulse frequency (50–250 Hz), treatment time (50–2050 µs), pulse width (1–7 µs), and pulse polarity (monopolar/bipolar). Vitamin C retention varied from 39.8% to 96.4%, depending on the PEF parameters composition used. Greater sensitivity to changes in PEF parameters was observed in the case of vitamin C than in the case of lycopene or antioxidant capacity of watermelon juice [119].

Similarly, the positive effects of the use of PEF were found by Elez-Martínez and Martín-Belloso [120], who treated orange juice and cold vegetable soup (*gazpacho*) with a high-intensity pulsed electric field (HIPEF). HIPEF-treated orange juice and *gazpacho* better-retained vitamin C (87.5–98.2% and 84.3–97.1%, respectively) than did heat-pasteurized products at 90 °C for 1 min did (82.4% and 79.2%, respectively). The authors showed that the retention of ascorbic acid strictly depends on the parameters of the PEF process. Bipolar pulses led to higher contents of vitamin C, and the tendency was as follows: The lower the electric field strength, shorter treatment time, lower pulse frequency and the pulse width, the higher vitamin C retention was. In another experiment, carrot juice was subjected to the pulsed electric field (bipolar, 35 kV/cm for 1500 µs, at 200 Hz) as well as to thermal treatment at 90 °C for 30 s or 60 s. Quitão-Teixeria et al. [121] demonstrated that PEF processing resulted in better vitamin C retention (95%) than heat treatments (87–89%). Moreover, the loss of vitamin C during the storage of carrot juice (for 56 days at 4 °C) was higher in thermally processed juices compared to PEF juice. Hence, vitamin C retention after storage of heat-treated juices was 14.1% (90 °C for 60 s) and 21.3% (90 °C for 30 s), whereas in the HIPEF treated carrot juice, it was 37.6% of its initial concentration.

Other authors in their studies also confirm a higher vitamin C retention in products preserved with PEF than in those subjected to traditional high-temperature pasteurization [122,123,124,125] and also during storage [126].

### 4.5. Minimally Processed Fruit and Vegetables (MPFV)

In many countries around the world, minimally processed fruit and vegetables are becoming an essential part of a healthy diet for people of all ages. The attractiveness of this market sector is primarily due to the convenience of fresh-cut/ready to eat food. The definition of “Fresh-cut produce” is any fresh fruit or vegetable that has been physically altered from its original form but remains in a fresh state. It has been trimmed, peeled, washed, and/or cut into a 100% usable product that is subsequently packed to offer consumers high nutrition, convenience, and value while still maintaining freshness [127].

The intensity of metabolic processes of fresh-cut produce is specific for species and cultivars and depends on the degree of raw material ripeness and technologies. Processing and storage temperature is the most important factor; however, the degree of disintegration also has a high impact on product respiration intensity and changes in highly sensitive products to oxidation such as vitamin C but also affects changes in other vitamins [128].

The whole fresh fruit and vegetables are richer in ascorbic acid than fresh-cut products, and additionally, higher reductions in vitamin C contents were found in fresh-cut vegetables than fruit [129]. Concerning market development safety of minimally processed products is the most important issue; therefore, food safety must be implemented in production standards. Washing and disinfection of fresh-cut fruit and vegetables before packing must be carried out [130]. Vanderkinderen et al. [131] found that water rinsing significantly decreased the vitamin C (maximum 35%) in fresh-cut iceberg lettuce, and that additional protective effects caused by adding a sanitizer such as sodium hypochlorite (20 and 200 mg/L), alternative sanitizers such as peroxyacetic acid (80 and 250 mg/L) and neutral electrolyzed oxidizing water (4.5 and 30 mg/L free chlorine) as well as chlorine dioxide, to the washing water, were not observed for vitamin C. The same group of scientists [132] working on ready-to-eat white cabbage found that the mechanical effects caused by washing with water already reduced the vitamin C content by 16–29%. Contrary to washing with neutral electrolyzed oxidizing water and contact with chlorine dioxide gas, a supplementary decrease of the vitamin C content ranging between 9% and 28% was observed when peroxyacetic acid or 200 mg/L sodium hypochlorite were used. Another experiment with fresh-cut bell pepper concluded that water-soluble nutrient contents of fresh-cut produce, such as ascorbic acid, decrease during a decontamination process that uses chlorine-based solutions according to the free chlorine concentration and the soaking time. A decrease in the L-ascorbic acid ratio to 80% was observed within 5 min of soaking in deionized water. The slightly acidic electrolyzed water treatment comparatively maintained L-ascorbic acid content than the sodium hypochlorite solution treatment [133].

In the case of leafy vegetables, characterized by a large surface, greater losses may be observed due to cutting and washing procedures. Cocetta et al. [134] studied the effect of cutting, temperature, and storage time on ascorbic acid in fresh-cut spinach leaves. Results measured in 24 h after processing and storage at 4 °C showed that for the control sample, L-ascorbic acid content was 19.4 mg/100 g, whereas for the cut one, it was 10 mg/100 g. After six days, the ascorbic acid levels were 10 mg/100 g in controls and 5 mg/100 g of fresh weight in cut leaves. The authors concluded that reduction in ascorbic acid was primarily affected by storage temperature and aggravated by cutting procedures.

To preserve ascorbic acid in minimally processed products, and the addition of several antioxidants was examined. Soaking of coleslaw in a solution of ascorbic acid and citric acid had a positive effect on the ascorbic acid content of the product. The best retention of ascorbic acid in the coleslaw mix stored for 12 days at 4 °C was found if the product was packed in the atmosphere with reduced oxygen contents [135]. Immersing escarole in 1% ascorbic acid and especially in 1% citric acid solution contributed to the highest retention of endogenous ascorbic acid during 21 days of escarole storage at 0 °C [136].

To minimize microbial hazards of minimally processed fruit and vegetable besides washing raw material in water with the addition of disinfectant, also treatment with UV and ozone are practiced. It was found that treatment with UV-C ultraviolet radiation (100-280 nm; 10, 20, and 30 kJ/m^2^) and gaseous ozone (1, 5, and 10 mg/L) had no adverse effect on vitamin C in minimally processed rocket leaves during 12 days of storage at 5 °C [137].

## 5. Effect of Innovative Processing on Vitamin C Bioavailability 

Most fruits and vegetables are an excellent source of bioactive compounds that have strong health-promoting properties. However, not only the high level of these ingredients in food is an important health impact factor. Nowadays, people know that the essence is the bioavailability of nutrients and whether the ingested nutrients are effectively absorbed and will appear in the blood plasma. Disruption of the natural matrix or the microstructure created during processing may influence the release, transformation, and subsequent absorption of some nutrients in the digestive tract [138]. The concept of bioaccessibility can be defined as the quantity or fraction, which is released from the food matrix in the gastrointestinal tract and becomes available for absorption [139]. The bioavailability of nutrients depends on many factors, including physicochemical properties of nutraceuticals, kind of food matrix, processing, and storage condition [54,140,141]. For example, the action of ultrasound has increased the bioavailability of the proteins from the cereals (rice, oat, corn, and soy) [140]. These effects are defined as “matrix effects” that can change the bioactive properties, bioaccessibility, and bioavailability of nutrients, playing an important role in designing functional foods [141].

The more and more frequently used innovative technologies, e.g., HPP and PEF, by the influencing on the structure of fruit and vegetables, allow increasing the bioavailability of bioactive ingredients, such as lycopene in tomatoes, phenolic compounds in apples, carotenes in carrots, starch, microelements, and amino acids in the germinated brown rice [141].

There are still very scanty reports in the literature showing the effects of the application of fruit and vegetable processing technology on the bioavailability of vitamin C. In recent years, a team of researchers in Spain has investigated the relationship between the use of innovative non-thermal processing technologies and the bioavailability of vitamin C from processed fruits and vegetables, taking into account clinical trials on the human population in their work [142,143,144,145,146].

Sánchez-Moreno and colleagues [142] conducted studies to check the bioavailability of vitamin C and the level of 8-isoprostane (8-epiPGF_2α_) after the consumption of PEF-treated (35 kV/cm, bipolar 4 µs pulse width at 800 Hz frequency, for 750 µs treatment time) orange juice compared to freshly squeezed (FS) one in a healthy human population. The research was carried out on two groups: 1) six volunteers who consumed 500 mL of PEF-treated orange juice and 2) six volunteers who drank 500 mL of FS orange juice in one dose during the first day and in two doses (250 mL in the morning and 250 mL in the afternoon) for 2–14 consecutive days. Studies have shown that drinking two glasses of the PEF-treated orange juice was associated with a significant increase in plasma vitamin C concentration and a decrease of 8-epiPGF_2α_ in plasma. Moreover, these effects were similar to those obtained with FS orange juice. This research demonstrates the potential of PEF technology to preserve orange juice because the juice retains the vitamin C bioavailability characteristics and the antioxidant properties of fresh juice, in addition to longer shelf life. A similar study was carried out on vegetable soup “gazpacho”, where the bioavailability of vitamin C from product subjected to pulsed electric fields (PEFs) and a freshly prepared (FM) soup and its effect on the concentration of 8-epiPGF_2α_ in the human population were evaluated. It was indicated that drinking two servings (500 mL) of PEF-treated or FM gazpacho daily increased plasma vitamin C and significantly decreases 8-epiPGF_2α_ concentrations in healthy humans. To summarize, the application of PEF as a preservation technique does not change the health properties of the processed product compared to the fresh product [143]. 

The same team of scientists also tested the bioavailability of vitamin C from orange juice, which was fixed using another non-thermal method—high-pressure processing [144]. The clinical trial layout was the same as for trials described in [142,143]. The authors examined the bioavailability of vitamin C and its effects on plasma levels of vitamin C, uric acid (UA), F2-isoprostanes (8-epiPGF_2α_), C-reactive protein (CRP), and prostaglandin E2 (PGE2) in a healthy human population. Studies have confirmed that drinking HP-treated orange juice increases plasma vitamin C and decreases 8-epiPGF_2α_ and PGE2 levels in humans, which may help reduce the risk of chronic diseases. 

The study regarding vitamin C bioavailability was carried out in two steps, a dose-response and a multiple-dose response test. For the dose-response measurement, the subjects were asked to consume 500 mL of juice or gazpacho after a minimum of 12 h of fasting, and blood samples were drawn before and every 60 min for 6 h. For the multiple dose-response, the subjects were instructed to drink the juice or gazpacho at home, in two doses, 250 mL in the morning and 250 mL in the afternoon, for two consecutive weeks. Blood samples were taken again during the intervention on the seventh and fourteenth day of the study. The maximum increase in plasma vitamin C occurred 3–4 h post-dose in the HPP as well as PEF-treated groups for both products. Compared with the baseline, the vitamin C concentration was significantly higher on day seven and 14 of the intervention. It was also stated that consuming PEF-treated products daily caused an increase in plasma vitamin C, improved the vitamin C bioavailability, and provided a longer shelf-life of fresh products, in addition, to a decline in oxidative stress in healthy humans [145].

In another study, the three kinds of beverages were subjected to different methods of conservation (high-intensity pulsed electric fields (HIPEF), high-pressure processing (HPP), and thermal treatment (TT)). The bioaccessibility of vitamin C was analyzed using an in vitro method. This method consisted of two sequential stages: gastric (pH 2, containing pepsin) and small intestinal digestions with dialysis (pH 7, containing a pancreatin-bile mixture). The treatment by non-thermal processing (HIPEF and HPP) did not modify the bioaccessibility of vitamin C in comparison with untreated beverages. In contrast, significant losses in the vitamin C bioaccessibility were observed in thermally treated (90 °C for 60 s) beverages. The authors explained this effect by the fact that HIPEF and HPP technologies are able to inactivate some oxidative enzymes, avoiding the oxidation of vitamin C and thus maintaining its active form and bioaccessibility in beverages treated by these methods [146].

In the review [141], which analyzed the impact of PEF on the bioavailability and bioaccessibility of bioactive compounds, the authors confirmed that the developing trend of using non-thermal methods in the food industry has a positive effect on the bioavailability of health-related ingredients, including vitamin C. At the same time, they emphasized the need for conducting further research focusing on the non-thermal processing of different fruits and vegetables with their impact on human health in addition to their bioavailability [147].

## 6. Conclusions

Fruit and vegetables are the major sources of vitamin C irrespectively of the form—fresh or processed; however, fruit and fruit juices are usually contribute more to ascorbic acid intake than vegetables. As stated in Dietary Guidelines for Americans “all forms of foods, including fresh, canned, dried, and frozen, can be included in healthy eating patterns”.

High-pressure processing was shown to preserve vitamin C in fruit and vegetable juices at conditions required to achieve a 5-log reduction of pathogenic microorganisms. Pulsed electric field technology is very beneficial regarding retention of the main health-promoting compounds, including vitamin C, and it has a good perspective for commercial implementation. Ultrasounds increase the bioavailability of vitamin C in fruit juices, but on the other hand, they intensify the degradation of ascorbic acid during the US-assisted drying of fruit and vegetables.

Nevertheless, the use of each technology requires careful adjustment of the process parameters depending on the kind of processed raw material to protect against excessive losses of vitamin C. Studies on the application of non-thermal technologies in food processing have shown their effectiveness in increasing the nutritional value, including the preservation of vitamin C in processed fruits and vegetables. The development of innovative processing technologies not only contributes to the production of safer but also with higher nutritional and health-promoting properties food, due to increasing the bioavailability of bioactive compounds, including vitamin C, which is necessary for the proper functioning of our body.

## Figures and Tables

**Table 1:** L-Ascorbic acid content in fresh fruit [12].

Fruit Species	Content mg/100 g	Fruit Species	Content mg/100 g
Blackurrants	181.0	Blackberries	21.0
Green kiwi fruits	92.7	Quinces	15.0
Pummelo	61.0	Cranberries	14.0
Papayas	60.9	Pomegranates	10.2
Oranges, navels	59.1	Apricots	10.0
Strawberries	58.8	Red sour cherries	10.0
Oranges, common variety	53.2	Avocados	10.0
Lemons, without peel	53.0	Blueberries	9.7
Pineapple	47.8	Plums	9.5
Oranges, Florida	45.0	Bananas	8.7
Red and white currants	41.0	Persimmons, Japanse	7.5
Mangos	36.4	Sweet cherries,	7.0
Elderberries	36.0	Yellow peaches,	6.6
White grapefruit,	33.3	Grapes, Muscadine	6.5
Pink and red grapefruit	31.2	Nectarines	5.4
Limes	29.1	Apples, with skin	4.6
Gooseberries	27.7	Pears	4.3
Tangerines, (mandarin)	26.7	Apples, without skin	4.0
Raspberries	26.2	Red or green Grapes	3.2

Note: Figures in violet depict the amount of ascorbic acid above 100% of European Union (EU) reference value (RWS). Those marked in red between 30% and 100%, and those in green between 15% and 30%.

**Table 2:** L-Ascorbic acid content in fresh vegetables [12].

Vegetable Species	Content mg/100 g	Vegetable Species	Content mg/100 g
Green peppers, hot chili	242.5	Beet greens	30.0
Red peppers, hot chili	143.7	New Zealand spinach	30.0
Parsley	133.0	Green soybeans	29.0
Red peppers, sweet	127.7	Spinach	28.1
Kale	120.0	Cabbage, Chinese (Pe-Tsai),	27.0
Broccoli	89.2	Rutabagas raw	25.0
Green cauliflower	88.1	Green chicory	24.0
Brussels sprouts	85.0	Green tomatoes	23.4
Green peppers, sweet	80.4	Turnips	21.0
Kohlrabi	62.0	Potatoes, flesh, and skin	19.7
Turnip greens	60.0	Melons, honeydew	18.0
Peas, edible-podded	60.0	Squash, summer, zucchini	17.9
Chives	58.1	Parsnips	17.0
Red cabbage	57.0	Squash, summer	17.0
Cabbage, common, freshly harvested	51.0	Yellow beans, snap	16.3
Cauliflower	48.2	Orange tomatoes	16.0
Cabbage, chinese (Pak-Choi)	45.0	Arugula	15.0
Green peas	40.0	Radishes	14.8
Melons, cantaloupe	36.7	Red Tomatoes, ripe	13.7
Cabbage	36.6	Squash, winter, all varieties,	12.3
Squash, zucchini, baby	34.1	Green beans, snap	12.2
Broadbeans, immature seeds	33.0	Leeks (bulb and lower leaf-portion)	12.0
Cabbage, Savoy	31.0	Lettuce, green leaf	9.2

Note: Figures in violet depict the amount of ascorbic acid above 100% of EU reference value (RWS). Those marked in red between 30% and 100%, and those in green between 15% and 30%.
